# Laparoscopic proximal gastrectomy with double‐flap technique *versus* laparoscopic subtotal gastrectomy for proximal early gastric cancer

**DOI:** 10.1002/bjs5.50241

**Published:** 2019-12-12

**Authors:** Y. Kano, M. Ohashi, S. Ida, K. Kumagai, T. Sano, N. Hiki, S. Nunobe

**Affiliations:** ^1^ Department of Gastroenterological Surgery, Gastroenterological Centre Cancer Institute Hospital, Japanese Foundation for Cancer Research 3‐8‐31 Ariake, Koto‐ku, Tokyo 135‐8550 Japan

## Abstract

**Background:**

Laparoscopic proximal gastrectomy with double‐flap technique (LPG‐DFT) and laparoscopic subtotal gastrectomy (LSTG) may replace laparoscopic total gastrectomy (LTG) for proximal early gastric cancer. The aim of this study was to evaluate short‐ and long‐term outcomes after LPG‐DFT and LSTG.

**Methods:**

Patients who underwent LPG‐DFT or LSTG at the Cancer Institute Hospital in Tokyo between January 2006 and April 2015 were included in this retrospective study. Operative procedures were selected based on the distance from the cardia to the proximal boundary of the tumour, tumour location and predicted remnant stomach volume. Patient characteristics, surgical data, markers of postoperative nutritional status, such as blood chemistry and bodyweight loss, and endoscopic findings were compared between procedures. The main study outcome was nutritional status.

**Results:**

A total of 161 patients (LPG‐DFT 51, LSTG 110) were included. Types of postoperative complication occurring more than 30 days after surgery differed between the two procedures. Remnant stomach ulcers, including anastomotic ulcers, were observed only after LPG‐DFT, whereas complications involving the small intestine, such as internal hernia or small bowel obstruction, occurred more frequently after LSTG. Values for total protein, albumin, prealbumin and bodyweight loss were comparable between the two procedures at 36 months after surgery. Haemoglobin concentrations were higher after LPG‐DFT than after LSTG at 24 months (13·4 *versus* 12·8 g/dl respectively; *P* = 0·045) and 36 months (13·5 *versus* 12·8 g/dl; *P* = 0·007) after surgery. The rate of Los Angeles grade B or more severe reflux oesophagitis was comparable.

**Conclusion:**

LPG‐DFT and LSTG for proximal early gastric cancer have similar outcomes, but different types of complication.

## Graphical abstract

Laparoscopic proximal gastrectomy with double‐flap technique is different from laparoscopic subtotal gastrectomy in terms of preventing problems involving the small intestine and anaemia after surgery for proximal early gastric cancer. However, these procedures are comparable for other outcomes, even though they are opposing procedures.

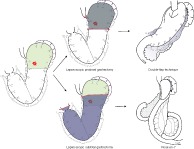

'Opposite techniques but both applicable'

## Abstract (es)

**Antecedentes:**

La gastrectomía proximal laparoscópica con técnica de doble derivación (*double flap technique*, LPG‐DFT) y la gastrectomía subtotal laparoscópica (*laparoscopic subtotal gastrectomy*, LsTG) pueden sustituir a la gastrectomía total laparoscópica (laparoscopic total gastrectomy, LTG) para el cáncer gástrico precoz (*early gastric cancer*, EGC) proximal. El objetivo de este estudio fue evaluar los resultados a corto y a largo plazo tras LPG‐DFT y LsTG.

**Métodos:**

En este estudio retrospectivo se incluyeron pacientes que fueron sometidos a LPG‐DFT o LsTG en el Hospital del Instituto del Cáncer de Tokio entre enero 2006 y abril 2015. Las técnicas quirúrgicas se seleccionaron en base a la distancia entre el cardias y el borde proximal del tumor, localización del tumor, y el volumen previsto del remanente gástrico. Las características de los pacientes, datos quirúrgicos, marcadores del estado nutricional postoperatorio, tales como la bioquímica sanguínea y la pérdida de peso corporal (*body weight loss*, BWL), y los hallazgos endoscópicos se compararon entre las técnicas. El resultado principal del estudio fue el estado nutricional.

**Resultados:**

Se incluyeron un total de 161 pacientes (LPG‐DFT 51, LsTG 110). Los tipos de complicaciones postoperatorias que aparecieron a más de 30 días después de la cirugía variaron entre ambas técnicas. Las úlceras en el remanente gástrico, incluyendo úlceras anastomóticas, solo se observaron tras una LPG‐DFT, mientras las complicaciones relacionadas con el intestino delgado, como la hernia interna o la obstrucción de intestino delgado, sucedió con más frecuencia tras una LsTG. Los valores de proteínas totales, albúmina, prealbúmina, y BWL fueron comparables entre ambas técnicas a los 36 meses después de la cirugía. Las concentraciones de hemoglobina fueron más altas tras una LPG‐DFT que tras una LsTG a los 24 (13,4 *versus* 12,8 mg/dL, *P* = 0,045) y 36 meses (13,5 *versus* 12,8 mg/mL, *P* = 0,007) después de la cirugía. Las incidencias de esofagitis por reflujo grado B Los Angeles o más grave fueron comparables.

**Conclusión:**

La LPG‐DFT y la LsTG para el EGC proximal presentan resultados bastante similares, pero difieren en el tipo de complicaciones.

## Introduction

The incidence of upper‐third gastric cancer, including early gastric cancers, is increasing in Korea, China and Japan^1^, ^2^, ^3^. Laparoscopic total gastrectomy (LTG), laparoscopic proximal gastrectomy (LPG) and laparoscopic subtotal gastrectomy (LSTG) are all technically feasible operative procedures for such lesions. In recent studies^4^, ^5^, ^6^, better surgical outcomes, including nutritional status, bodyweight loss and quality of life were reported after LPG compared with LTG. The mechanism, however, is unclear.

Surgical outcomes of LPG may depend on the type of reconstruction, as reflux oesophagitis is one of the most important determinants of long‐term outcome^7^, ^8^, ^9^, ^10^. The size of the remnant stomach may influence other long‐term outcomes, such as haemoglobin concentration.

LPG with double‐flap technique (LPG‐DFT) is currently one of the preferred reconstruction techniques for LPG in Japan. LPG‐DFT has better outcomes than LTG in terms of morbidity, postoperative hospital stay, reflux oesophagitis and postoperative nutritional status^11^. Although LPG‐DFT requires a more complex intracorporeal suturing technique and longer duration of surgery^12^, ^13^, good physiological function is maintained because of the relatively large remnant stomach and distinctive anastomotic technique for oesophagogastrostomy. This minimizes subsequent reflux oesophagitis, which may influence food intake, bodyweight, haemoglobin concentration and nutritional status. With previously reported comparable survival^14^, LPG‐DFT may be superior to LSTG.

The aim of the present study was to assess short‐ and long‐term outcomes of LPG‐DFT *versus* LSTG to determine the preferred procedure for resection of early gastric cancer in the proximal stomach.

## Methods

This was a retrospective study of consecutive patients who underwent LPG‐DFT or LSTG for cT1 N0 M0 gastric cancer in the upper third of the stomach at the Cancer Institute Hospital, Tokyo, Japan, between January 2006 and April 2015. Data were retrieved from a prospectively developed database. DFT reconstruction has been applied to LPG since January 2013. Before 2013, LTG had often been performed in patients with this disease, rather than LPG. Patients undergoing additional surgery after endoscopic mucosal dissection (ESD) were included in the study. Those who had tumours involving the oesophagus, synchronous cancer, metachronous cancer after surgery, or relapse were excluded. Clinical stage was classified according to the 14th edition of the Japanese Classification of Gastric Carcinoma^15^.

The study was approved by the Institutional Review Board of the Cancer Institute Hospital.

### Selection of surgical procedure

LSTG was initially intended to be performed in patients who fulfilled the following criteria: early gastric cancer diagnosed as cT1 N0; tumour located in or involving the upper third of the stomach, but not the fornix; and oral side of the tumour more than 3 cm (2 cm if tumour was located at the lesser curvature) from the oesophagogastric junction. If tumour located in the upper third of the stomach was not eligible for LSTG, an LTG or LPG‐DFT procedure was alternatively planned according to the treatment era. LPG‐DFT was planned when the size of the remnant stomach was estimated to be more than half that of the original stomach.

### Surgical procedures

#### Laparoscopic proximal gastrectomy with double‐flap technique *(Fig*. 1 *)*

**Figure 1:**
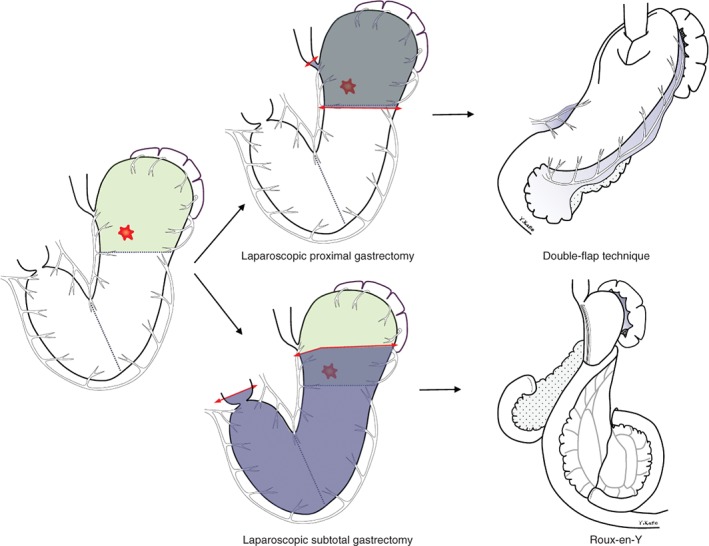
Resection and reconstruction in laparoscopic subtotal gastrectomy and laparoscopic proximal gastrectomy with double‐flap technique

In LPG‐DFT, D1+ lymphadenectomy was performed according to version 4 of the Japanese Gastric Cancer Treatment Guidelines^16^. After lymph node dissection, the oesophagus and stomach were transected under intraoperative endoscopic guidance using an endoscopic linear stapler. Intraoperative frozen‐section analysis of the distal, or sometimes proximal, surgical margin was performed in all patients except those who had undergone ESD. After transection of the oesophagus and stomach, oesophagogastrostomy was performed, based on Kamikawa's open surgical method^17^ and modified laparoscopic procedures reported previously^11^.

Briefly, double flaps were created extracorporeally by dissecting between the submucosal and muscular layers on the anterior wall of the remnant stomach. After creating the seromuscular double flaps, the walls of the oesophagus and gastric mucosa were sutured under laparoscopic view and an oesophagogastrostomy was created. The hinged flaps were used laparoscopically to cover the anastomosis and lower oesophagus.

#### Laparoscopic subtotal gastrectomy *(Fig*. 1 *)*

D1+ lymphadenectomy was performed in LSTG^16^; details of this surgical procedure have been described previously^18^. During lymph node dissection, several distal branches of the short gastric artery were divided, preserving at least one branch near the cardia. Depending on tumour location, the posterior gastric artery was sometimes divided. Intraoperative gastroscopy was performed to confirm the location of the tumour and marking clips were placed before surgery. In some patients in whom the boundary of the tumour was very close to the cardia and/or fornix, cautery markings were made by endoscopy on the day before surgery for use as landmarks instead of clips^19^. The stomach was transected with an endoscopic linear stapler, leaving a very small remnant stomach. Intraoperative frozen‐section analysis of the proximal surgical margin was performed in all patients. After lymph node dissection, a Roux‐en‐Y reconstruction was created via an antecolic route. Gastrojejunostomy was performed using an endoscopic linear stapler or a 25‐mm circular stapler with a perorally inserted anvil (Orvil®; Covidien, Mansfield, Massachusetts, USA). A side‐to‐side jejunostomy was performed approximately 30–40 cm distal from the gastrojejunal anastomosis. Since 2010, after creation of the anastomosis the Petersen defect has been closed with a non‐absorbable suture.

### Outcomes

Clinical, surgical and pathological variables were recorded. The severity of postoperative complications was evaluated according to the Clavien–Dindo classification^20^, ^21^. Early postoperative complications were defined as Clavien–Dindo grade II or above, occurring within 30 days of surgery. Late postoperative complications that required surgical or radiological intervention, or drug treatment, more than 30 days after surgery were regarded as an adverse event. Indicators of nutritional status, such as total protein, serum albumin, prealbumin and haemoglobin levels, were evaluated at 1, 3, 6, 12, 24 and 36 months after surgery. Bodyweight loss was evaluated at 12, 24 and 36 months. Reflux oesophagitis was assessed by endoscopic examination at 12 and 36 months, according to the Los Angeles classification^22^; that of grade B or above was recorded. The degree of food residue was evaluated according to the RGB (residue, gastritis, bile) classification, and the rate of residue of grade 2 or above calculated^23^.

Survival outcomes were not evaluated in this study. The main outcome was nutritional status.

### Statistical analysis

Baseline characteristics and outcomes of LPG‐DFT and LSTG were compared. Categorical variables were analysed using Fisher's exact test. Continuous variables were analysed with the Mann–Whitney *U* test. All statistical analyses were performed with SPSS® version 25 (IBM, Armonk, New York, USA). *P* < 0·050 was considered to denote statistical significance.

## Results

**Figure 2:**
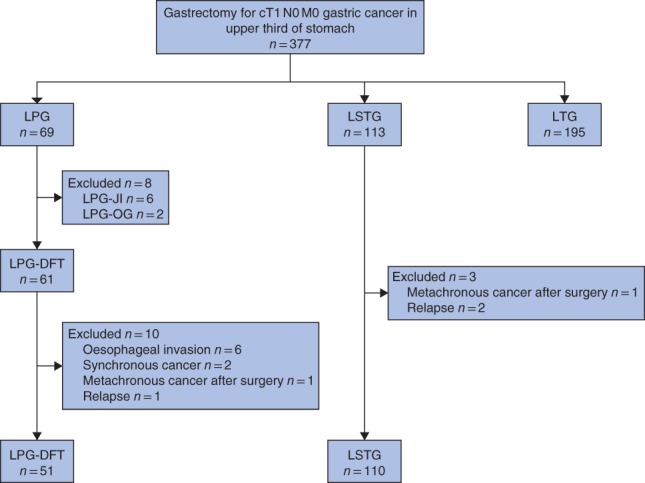
Flow diagram for the study LPG, laparoscopic proximal gastrectomy; LSTG, laparoscopic subtotal gastrectomy; LTG, laparoscopic total gastrectomy; LPG‐JI, laparoscopic proximal gastrectomy with jejunal interposition reconstruction; LPG‐OG, laparoscopic proximal gastrectomy with oesophagogastric anastomosis; LPG‐DFT, laparoscopic proximal gastrectomy with double‐flap technique.

A total of 377 patients with cT1 N0 M0 gastric cancer in the upper third of the stomach underwent LPG (69 patients), LSTG (113) or LTG (195) between January 2006 and April 2015. After exclusions, of patients who had undergone LPG or LSTG, 51 who had LPG‐DFT and 110 who underwent LSTG were eligible for inclusion in the study (*Fig*. 2).

Patient characteristics are summarized in *Table* 1. Patients who had LSTG were significantly younger than those in the LPG‐DFT cohort. The pathological margin was greater for LPG‐DFT than for LSTG. More patients in the LPG‐DFT than in the LSTG group required additional surgery after ESD. No significant differences were observed between the groups regarding sex, BMI, tumour size, pathological stage or adjuvant chemotherapy.

**Table 1:** Patient characteristics according to surgical procedure

	LPG‐DFT (*n* = 51)	LSTG (*n* = 110)	*P* †
**Age (years)** *	71 (61–77)	63 (55–69)	0·002‡
**Sex ratio (M : F)**	36 : 15	77 : 33	1·000
**BMI (kg/m** ^**2**^ **)** *	23·2 (21·0–25·2)	22·7 (20·6–24·7)	0·328‡
**Additional surgery after ESD**	19 (37)	20 (18·2)	0·011
**Tumour size (mm)** *	25 (17·5–38·5)	25 (18–38·7)	0·996‡
**Pathological margin (mm)** *	26 (20·5–36·0)	13 (8·3–18·0)	< 0·001‡
**Pathological stage**			0·204
IA	38 (75)	92 (83·6)	
IB	8 (16)	14 (12·7)	
IIA	2 (4)	3 (2·7)	
IIB	3 (6)	1 (0·9)	
**Adjuvant chemotherapy**	3 (6)	1 (0·9)	0·092

Values in parentheses are percentages unless indicated otherwise;

* values are median (i.q.r.). LPG‐DFT, laparoscopic proximal gastrectomy with double‐flap technique; LSTG, laparoscopic subtotal gastrectomy; ESD, endoscopic submucosal dissection.

† Fisher's exact test, except

‡ Mann–Whitney *U* test.

### Surgical data and postoperative complications

Surgical data and postoperative complications are shown in *Table* 2. The median duration of surgery was significantly shorter and intraoperative blood loss lower for LSTG *versus* LPG‐DFT. No difference in early postoperative complications was observed between the groups. Longer‐term complications varied between LPG‐DFT and LSTG: complications involving the remnant stomach were more common after LPG‐DFT, whereas those not involving the remnant stomach occurred more frequently after LSTG. Remnant stomach ulcers, including anastomotic ulcers, were observed only after LPG‐DFT. In contrast, internal hernia, small bowel obstruction and cholecystitis were seen only after LSTG. Anastomotic stricture developed in four patients (8 per cent) who had undergone LPG‐DFT and in three (2·7 per cent) who had undergone LSTG. All such strictures were treated successfully by endoscopic balloon dilatation. All patients who developed an anastomotic stricture after LSTG had undergone gastrojejunostomy using a circular stapler, whereas no strictures developed in side‐to‐side anastomoses created with a linear stapler.

**Table 2:** Surgical outcomes and postoperative complications according to operative procedure

	LPG‐DFT (*n* = 51)	LSTG (*n* = 110)	*P* †
**Duration of surgery (min)** *	404 (339–446)	289 (233–325)	< 0·001‡
**Blood loss (ml)** *	68 (29–120)	30 (20–70)	0·007‡
**Early postoperative complications**			
≥ Grade III	3 (6)	9 (8·2)	0·754
≥ Grade II	5 (10)	25 (22·7)	0·053
Anastomotic leakage	0 (0)	0 (0)	
Anastomotic bleeding	0 (0)	3 (2·7)	
Abdominal abscess	2 (4)	6 (5·5)	
Pancreatic fistula	0 (0)	1 (0·9)	
Intra‐abdominal infection	0 (0)	3 (2·7)	
Small bowel obstruction	1 (2)	2 (1·8)	
Enteritis	1 (2)	4 (3·6)	
Other	1 (2)	6 (5·5)	
**Mortality**	0 (0)	0 (0)	1·000
**Late postoperative complications**	8 (16)	15 (13·6)	1·000
Anastomotic stricture	4 (8)	3 (2·7)	
Anastomotic ulcer	1 (2)	0 (0)	
Remnant stomach ulcer	3 (6)	0 (0)	
Internal hernia	0 (0)	4 (3·6)	
Cholecystitis	0 (0)	4 (3·6)	
Small bowel obstruction	0 (0)	3 (2·7)	
Abscess	0 (0)	1 (0·9)	

Values in parentheses are percentages unless indicated otherwise;

* values are median (i.q.r.). LPG‐DFT, laparoscopic proximal gastrectomy with double‐flap technique; LSTG, laparoscopic subtotal gastrectomy.

† Fisher's exact test, except

‡ Mann–Whitney *U* test.

### Postoperative nutritional status

**Figure 3:**
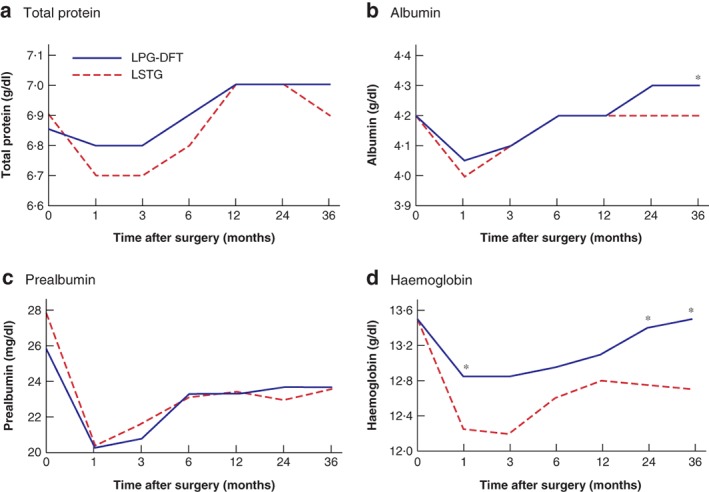
Comparison of nutritional status after laparoscopic proximal gastrectomy with double‐flap technique and laparoscopic subtotal gastrectomy with a very small remnant stomach **a** Total protein, **b** albumin, **c** prealbumin and **d** haemoglobin concentrations following laparoscopic proximal gastrectomy with double‐flap technique (LPG‐DFT) and laparoscopic subtotal gastrectomy (LSTG). *P < 0·050 *versus* LSTG (Mann–Whitney *U* test).

**Figure 4:**
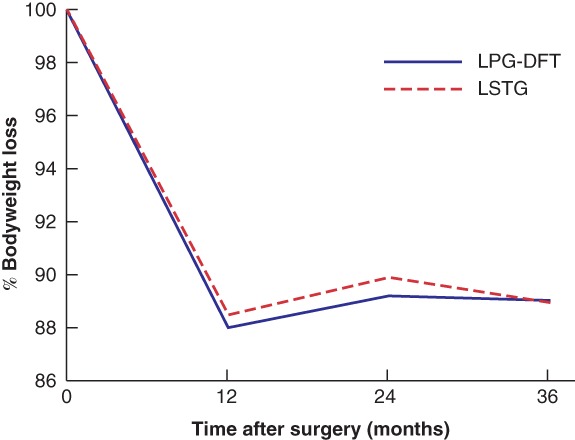
Bodyweight loss after surgery LPG‐DFT, laparoscopic proximal gastrectomy with double‐flap technique; LSTG, laparoscopic subtotal gastrectomy.

Nutritional status after surgery after LPG‐DFT and LSTG is shown in *Fig*. 3. Indicators of nutritional status, such as total protein, albumin and prealbumin concentrations, did not differ significantly between LPG‐DFT and LSTG groups. The albumin concentration at 36 months after surgery was higher after LPG‐DFT than after LSTG (4·3 *versus* 4·2 g/dl respectively; *P* = 0·028). Haemoglobin concentration was significantly higher after LPG‐DFT than after LSTG at 1 month (12·9 *versus* 12·3 g/dl; *P* = 0·009), 24 months (13·4 *versus* 12·8 g/dl; *P* = 0·045) and 36 months (13·5 *versus* 12·8 g/dl; *P* = 0·007) after surgery (*Fig*. 3). In contrast, bodyweight loss did not differ between LPG‐DFT and LSTG groups (*Fig*. 4).

### Endoscopic findings

Endoscopic findings 12 and 36 months after surgery are shown in *Table* 3. Reflux oesophagitis of Los Angeles grade B or higher was observed in one and two patients at 12 and 36 months respectively after LPG‐DFT. Reflux oesophagitis improved after administration of a proton pump inhibitor. One patient had reflux oesophagitis 12 months after LSTG.

**Table 3:** Endoscopic findings 12 and 36 months after surgery according to operative procedure

	LPG‐DFT (*n* = 51)	LSTG (*n* = 110)	*P* ‡
**Reflux oesophagitis**			
At 12 months	1 (2)	0 of 100 (0)*	0·317
At 36 months	2 (4)	1 of 104 (1·0)†	0·236
**Residual foods**			
At 12 months	1 (2)	0 (0)	0·317
At 36 months	1 (2)	0 (0)	0·317

Values in parentheses are percentages. Endoscopy was not performed in *ten and †six patients. LPG‐DFT, laparoscopic proximal gastrectomy with double‐flap technique; LSTG, laparoscopic subtotal gastrectomy.

‡ Fisher's exact test.

## Discussion

Although LPG‐DFT and LSTG are opposing procedures for early gastric cancer in the proximal stomach, short‐ and long‐term outcomes are similar, with some differences in terms of late postoperative complications and postgastrectomy anaemia.

LPG‐DFT incorporates a simple reconstruction that does not involve the small intestine. Therefore, small intestine‐related complications, such as internal hernia or small bowel obstruction, are rare. This is an advantage of LPG‐DFT, because complications associated with the small intestine may be problematic after laparoscopic gastrectomy^4^, ^7^. Postoperative anastomotic stricture is another possible complication of proximal gastrectomy, especially after oesophagogastrostomy. Oesophagogastric anastomotic stricture occurred in 8 per cent of the patients, a lower rate than that reported in most other studies (range 8·2–28·0 per cent)^7^, ^24^, ^25^, ^26^, ^27^.

The size and location of the remnant stomach do not seem to influence the absorption of amino acids, but may significantly influence iron absorption, which requires ionization of iron by gastric acid secreted by the parietal cells. Almost all of the gastric body, in which many parietal and chief cells are located, and the area where the fundic glands are densely located, are resected in the LSTG procedure, whereas the LPG‐DFT procedure preserves more of the gastric body. Ingested food that is stored in the remnant stomach is mixed with acid more after LPG‐DFT than after the LSTG procedure, because food passes through the stomach in LSTG where there is no reservoir function. Haemoglobin concentrations in patients who had undergone LSTG decreased over time because of decreased iron uptake, iron storage or decreased vitamin B12 concentration. Iron absorption occurs mainly while food passes through the duodenum^28^, ^29^. Food passes through the duodenum in LPG‐DFT, but not in LSTG. All parietal cells, which are necessary for uptake of vitamin B12 in the terminal ileum, are resected in LSTG. Because iron and vitamin B12 concentrations were not measured in all patients, it is not clear what most influenced development of anaemia in the patients who had LSTG. The main problem following proximal gastrectomy is reflux oesophagitis, which was rare in both groups.

The present study has several limitations, including its retrospective nature and limited generalizability, as data are from a single centre. Symptoms were not assessed, although they are important in evaluating outcomes after gastrectomy. To evaluate postgastrectomy outcomes more precisely and compare outcomes between different procedures, assessment of symptoms using a questionnaire such as the Postgastrectomy Syndrome Assessment Scale 45 or the European Organization for Research and Treatment of Cancer Quality of Life Questionnaire C30 should be considered in prospective studies^30^, ^31^. Indications for performing LPG‐DFT and LSTG were different. No patient with disease located in the fundus or oesophagogastric junction had LSTG, although patients with disease in the proximal stomach could potentially undergo either LPG‐DFT or LPG.

## References

[1] Deans C , Yeo MS , Soe MY , Shabbir A , Ti TK , So JB . Cancer of the gastric cardia is rising in incidence in an Asian population and is associated with adverse outcome. World J Surg 2011; 35: 617–624. 21203759 10.1007/s00268-010-0935-0

[2] Zhou Y , Zhang Z , Zhang Z , Wu J , Ren D , Yan X *et al* A rising trend of gastric cardia cancer in Gansu Province of China. Cancer Lett 2008; 269: 18–25. 18501504 10.1016/j.canlet.2008.04.013

[3] Ahn HS , Lee HJ , Yoo MW , Jeong SH , Park DJ , Kim HH *et al* Changes in clinicopathological features and survival after gastrectomy for gastric cancer over a 20‐year period. Br J Surg 2011; 98: 255–260. 21082693 10.1002/bjs.7310

[4] Jung DH , Lee Y , Kim DW , Park YS , Ahn SH , Park DJ *et al* Laparoscopic proximal gastrectomy with double tract reconstruction is superior to laparoscopic total gastrectomy for proximal early gastric cancer. Surg Endosc 2017; 31: 3961–3969. 28342130 10.1007/s00464-017-5429-9

[5] Toyomasu Y , Ogata K , Suzuki M , Yanoma T , Kimura A , Kogure N *et al* Restoration of gastrointestinal motility ameliorates nutritional deficiencies and body weight loss of patients who undergo laparoscopy‐assisted proximal gastrectomy. Surg Endosc 2017; 31: 1393–1401. 27444825 10.1007/s00464-016-5127-z

[6] Ichikawa D , Komatsu S , Kubota T , Okamoto K , Shiozaki A , Fujiwara H *et al* Long‐term outcomes of patients who underwent limited proximal gastrectomy. Gastric Cancer 2014; 17: 141–145. 23558459 10.1007/s10120-013-0257-7

[7] Yasuda A , Yasuda T , Imamoto H , Kato H , Nishiki K , Iwama M *et al* A newly modified esophagogastrostomy with a reliable angle of His by placing a gastric tube in the lower mediastinum in laparoscopy‐assisted proximal gastrectomy. Gastric Cancer 2015; 18: 850–858. 25318978 10.1007/s10120-014-0431-6

[8] Tokunaga M , Ohyama S , Hiki N , Hoshino E , Nunobe S , Fukunaga T *et al* Endoscopic evaluation of reflux esophagitis after proximal gastrectomy: comparison between esophagogastric anastomosis and jejunal interposition. World J Surg 2008; 32: 1473–1477. 18264827 10.1007/s00268-007-9459-7

[9] Aburatani T , Kojima K , Otsuki S , Murase H , Okuno K , Gokita K *et al* Double‐tract reconstruction after laparoscopic proximal gastrectomy using detachable ENDO‐PSD. Surg Endosc 2017; 31: 4848–4856. 28389804 10.1007/s00464-017-5539-4

[10] Nakamura M , Nakamori M , Ojima T , Katsuda M , Iida T , Hayata K *et al* Reconstruction after proximal gastrectomy for early gastric cancer in the upper third of the stomach: an analysis of our 13‐year experience. Surgery 2014; 156: 57–63. 24799083 10.1016/j.surg.2014.02.015

[11] Hayami M , Hiki N , Nunobe S , Mine S , Ohashi M , Kumagai K *et al* Clinical outcomes and evaluation of laparoscopic proximal gastrectomy with double‐flap technique for early gastric cancer in the upper third of the stomach. Ann Surg Oncol 2017; 24: 1635–1642. 28130623 10.1245/s10434-017-5782-x

[12] Muraoka A , Kobayashi M , Kokudo Y . Laparoscopy‐assisted proximal gastrectomy with the hinged double flap method. World J Surg 2016; 40: 2419–2424. 27094564 10.1007/s00268-016-3510-5

[13] Kuroda S , Nishizaki M , Kikuchi S , Noma K , Tanabe S , Kagawa S *et al* Double‐flap technique as an antireflux procedure in esophagogastrostomy after proximal gastrectomy. J Am Coll Surg 2016; 223: e7–e13. 27157920 10.1016/j.jamcollsurg.2016.04.041

[14] Kano Y , Ohashi M , Ida S , Kumagai K , Nunobe S , Sano T *et al* Oncological feasibility of laparoscopic subtotal gastrectomy compared with laparoscopic proximal or total gastrectomy for cT1 N0 M0 gastric cancer in the upper gastric body. Gastric Cancer 2019; 22: 1060–1068. 30830641 10.1007/s10120-019-00947-7

[15] Japanese Gastric Cancer Association . Japanese classification of gastric carcinoma: 3rd English edition. Gastric Cancer 2011; 14: 101–112. 21573743 10.1007/s10120-011-0041-5

[16] Japanese Gastric Cancer Association. Japanese gastric cancer treatment guidelines 2014 (ver. 4). Gastric Cancer 2017; 20: 1–19. 10.1007/s10120-016-0622-4 PMC5215069 27342689

[17] Kamikawa Y , Kobayashi T , Kamiyama S , Satomoto K . [A new procedure of esophagogastrostomy to prevent reflux following proximal gastrectomy.] Shoukakigeka 2001; 24: 1053–1060.

[18] Jiang X , Hiki N , Nunobe S , Nohara K , Kumagai K , Sano T *et al* Laparoscopy‐assisted subtotal gastrectomy with very small remnant stomach: a novel surgical procedure for selected early gastric cancer in the upper stomach. Gastric Cancer 2011; 14: 194–199. 21347820 10.1007/s10120-011-0023-7

[19] Kamiya S , Ohashi M , Ida S , Kumagai K , Nunobe S , Sano T *et al* Laparoscopic subtotal gastrectomy with a new marking technique, endoscopic cautery marking: preservation of the stomach in patients with upper early gastric cancer. Surg Endosc 2018; 32: 4681–4687. 29922851 10.1007/s00464-018-6272-3

[20] Dindo D , Demartines N , Clavien PA . Classification of surgical complications: a new proposal with evaluation in a cohort of 6336 patients and results of a survey. Ann Surg 2004; 240: 205–213. 15273542 10.1097/01.sla.0000133083.54934.ae PMC1360123

[21] Clavien PA , Barkun J , de Oliveira ML , Vauthey JN , Dindo D , Schulick RD *et al* The Clavien–Dindo classification of surgical complications: five‐year experience. Ann Surg 2009; 250: 187–196. 19638912 10.1097/SLA.0b013e3181b13ca2

[22] Armstrong D , Bennett JR , Blum AL , Dent J , De Dombal FT , Galmiche JP *et al* The endoscopic assessment of esophagitis: a progress report on observer agreement. Gastroenterology 1996; 111: 85–92. 8698230 10.1053/gast.1996.v111.pm8698230

[23] Kubo M , Sasako M , Gotoda T , Ono H , Fujishiro M , Saito D *et al* Endoscopic evaluation of the remnant stomach after gastrectomy: proposal for a new classification. Gastric Cancer 2002; 5: 83–89. 12111583 10.1007/s101200200014

[24] Hosoda K , Yamashita K , Katada N , Moriya H , Mieno H , Shibata T *et al* Potential benefits of laparoscopy‐assisted proximal gastrectomy with esophagogastrostomy for cT1 upper‐third gastric cancer. Surg Endosc 2016; 30: 3426–3436. 26511124 10.1007/s00464-015-4625-8

[25] Nishigori T , Okabe H , Tsunoda S , Shinohara H , Obama K , Hosogi H *et al* Superiority of laparoscopic proximal gastrectomy with hand‐sewn esophagogastrostomy over total gastrectomy in improving postoperative body weight loss and quality of life. Surg Endosc 2017; 31: 3664–3672. 28078458 10.1007/s00464-016-5403-y

[26] Masuzawa T , Takiguchi S , Hirao M , Imamura H , Kimura Y , Fujita J *et al* Comparison of perioperative and long‐term outcomes of total and proximal gastrectomy for early gastric cancer: a multi‐institutional retrospective study. World J Surg 2014; 38: 1100–1106. 24310733 10.1007/s00268-013-2370-5

[27] Ahn SH , Lee JH , Park DJ , Kim HH . Comparative study of clinical outcomes between laparoscopy‐assisted proximal gastrectomy (LAPG) and laparoscopy‐assisted total gastrectomy (LATG) for proximal gastric cancer. Gastric Cancer 2013; 16: 282–289. 22821182 10.1007/s10120-012-0178-x

[28] Chin AC , Espat NJ . Total gastrectomy: options for the restoration of gastrointestinal continuity. Lancet Oncol 2003; 4: 271–276. 12732163 10.1016/s1470-2045(03)01073-8

[29] Yang YS , Chen LQ , Yan XX , Liu YL . Preservation *versus* non‐preservation of the duodenal passage following total gastrectomy: a systematic review. J Gastrointest Surg 2013; 17: 877–886. 23460248 10.1007/s11605-013-2174-9

[30] Nakada K , Ikeda M , Takahashi M , Kinami S , Yoshida M , Uenosono Y *et al* Characteristics and clinical relevance of postgastrectomy syndrome assessment scale (PGSAS)‐45: newly developed integrated questionnaires for assessment of living status and quality of life in postgastrectomy patients. Gastric Cancer 2015; 18: 147–158. 24515247 10.1007/s10120-014-0344-4

[31] Aaronson NK , Ahmedzai S , Bergman B , Bullinger M , Cull A , Duez NJ *et al* The European Organization for Research and Treatment of Cancer QLQ‐C30: a quality‐of‐life instrument for use in international clinical trials in oncology. J Natl Cancer Inst 1993; 85: 365–376. 8433390 10.1093/jnci/85.5.365

